# Ultrafast traveling wave dominates the electric organ discharge of *Apteronotus leptorhynchus*: an inverse modelling study

**DOI:** 10.1038/srep15780

**Published:** 2015-10-30

**Authors:** Aaron R. Shifman, André Longtin, John E. Lewis

**Affiliations:** 1 Department of Biology, University of Ottawa, Ottawa, Ontario K1N 6N5, Canada; 2 Center for Neural Dynamics, University of Ottawa, Ottawa, Ontario K1N 6N5, Canada; 3 Department of Physics, University of Ottawa, Ottawa, Ontario K1N 6N5, Canada

## Abstract

Identifying and understanding the current sources that give rise to bioelectric fields is a fundamental problem in the biological sciences. It is very difficult, for example, to attribute the time-varying features of an electroencephalogram recorded from the head surface to the neural activity of specific brain areas; model systems can provide important insight into such problems. Some species of fish actively generate an oscillating (c. 1000 Hz) quasi-dipole electric field to communicate and sense their environment in the dark. A specialized electric organ comprises neuron-like cells whose collective signal underlies this electric field. As a step towards understanding the detailed biophysics of signal generation in these fish, we use an anatomically-detailed finite-element modelling approach to reverse-engineer the electric organ signal over one oscillation cycle. We find that the spatiotemporal profile of current along the electric organ constitutes a travelling wave that is well-described by two spatial Fourier components varying in time. The conduction velocity of this wave is faster than action potential conduction in any known neuronal axon (>200 m/s), suggesting that the spatiotemporal features of high-frequency electric organ discharges are not constrained by the conduction velocities of spinal neuron pathways.

The production and detection of electric fields is essential for living organisms from the single-cell to humans^1^,^2^,^3^,^4^,^5^. Characterizing the unknown current sources underlying a particular bioelectric field is a problem faced in a variety of biological contexts, including the interpretation of electrocardiograms (ECGs), electroencephalograms (EEGs), as well as the brain recordings for deep-brain stimulation and brain-machine interfaces^6^. Weakly electric fish use a specialized electric organ to generate an electric field surrounding their body^7^,^8^. The so-called electric organ discharge (EOD) is the basis for a specialized active sense that enables navigation, communication and prey capture in the dark^8^,^9^. While the spatiotemporal aspects of these electric fields have been well-characterized^10^,^11^, relatively little is known about the electrical activity in the organ that generates the EOD.

The EODs of weakly electric fish come in two forms^9^. Pulse type fish generate pulses at irregular intervals, with average frequencies less that 150 Hz; the temporal waveform of the pulse can vary over longer time scales, but pulse duration is much shorter than the time between pulses^9^. In contrast, wave-type fish produce a very regular quasi-sinusoidal EOD within a species-specific frequency range from 200 Hz to 2 kHz^9^,^12^,^13^,^14^. The spatial nature of the resulting electric fields (both pulse and wave-type) involves a dipole-like geometry^12^,^15^,^16^. Various computational methods have been used to describe these fields (see reference^17^ for a recent review), along with the sensory consequences (field perturbations) due to complex objects^18^,^19^,^20^,^21^ and body position^11^,^22^. These models can also be used to explore the nature of signal generation by the electric organ itself^11^,^23^. Recently, Pedraja *et al.*^24^ made measurements of current flow at distinct points on the fish’s skin to constrain such a model. They described a diversity of spatiotemporal signal complexity across different species, while inferring the associated internal current sources. However, understanding the cellular-level biophysics which underlie these signals requires a detailed spatiotemporal description of electric organ current throughout the EOD cycle.

The electric organ (EO) comprises large populations of electrocytes that generate the current underlying the EOD. Electrocyte characteristics vary across species^12^,^25^,^26^. Myogenic EODs, which are found in most species, are generated by large electrocytes activated by spinal neurons via neuromuscular-like chemical synapses. Less is known, on the other hand, about high-frequency wave-type fish whose neurogenic EOD is generated by thin neuron-like electrocytes that are activated by spinal projection neurons via direct electrical connections^26^,^27^.

To better understand the biophysical mechanisms and constraints underlying high-frequency neurogenic EODs, we use a finite-element modelling (FEM) approach^18^ to (1) describe the spatiotemporal aspects of the electric field generated by *A. leptorhynchus*, a high-frequency (c. 1000 Hz) wave-type fish, and (2) determine the current sources produced by the electric organ. Previous studies^11^,^24^ have used the boundary element method (BEM) to model such fields in other species. The BEM model is efficient but restricts computations to current flux on the skin. Although computationally more demanding, the FEM approach allows us to predict the current density along the EO, within the body, as a function of time and space at a resolution that has not yet been achievable using other approaches. This level of detail allows us to show that EO activity comprises, as suggested previously, a travelling wave from head-to-tail^11^. We find that the conduction velocity of this wave exceeds the fastest known action potential conduction along myelinated spinal axons^28^,^29^. This suggests that the spatiotemporal features of high-frequency discharges are not constrained by the conduction velocities of spinal neurons. In future studies, our FEM approach can be integrated with detailed computational models of excitable cells to understand how the coordination of electrocyte action potentials leads to the high-frequency wave-like signalling involved in this active electric sense.

## Results

### The electric field model

**Figure 1:**
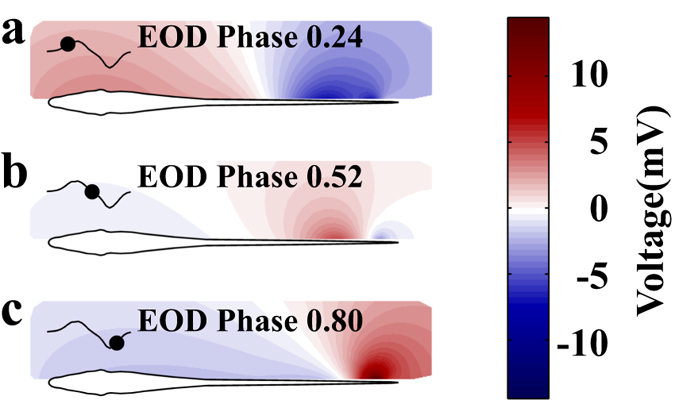
Summary of model output (voltage map) showing three stereotypic EOD field geometries in A.*leptorhynchus*. (**a**) Head-positive (EOD phase 0.24); normalized root-mean-squared error is 3.63%. (**b**) Intermediate phase (EOD phase 0.52) where head and distal tail regions are negative; normalized root-mean-squared error is 4.48%. (**c**) Head-negative (EOD phase 0.80); normalized root-mean-squared error is 3.13%.

The spatiotemporal electric field of *A. leptorhynchus* is complex relative to other weakly electric fish^11^. Previously, we have used a finite-element approach to model the electric potential produced by this fish in the horizontal plane, at a single phase of the oscillating electric organ discharge, EOD^18^,^30^. This 2D description proved accurate and useful for understanding various aspects of electrosensory processing, but was limited to a single snapshot in time. Here, we extend these models to include 25 different phases of the EOD cycle, based on data provided by Chris Assad and Brian Rasnow^10^,^11^ (see Methods for details). Our extended model captures the spatiotemporal complexities of the EOD, with good fits to the electric potential for all phases: the normalized root-mean-squared error (NRMSE) ranges from 2.55% to 5.86% (4.02% ± 0.92%; mean ± SD). Figure 1 shows the model output for three representative EOD phases. The so-called head-positive and head-negative phases show the typical elongated dipole-like field (Fig. 1a,c; EOD phases 0.24 and 0.8 respectively; phases defined on the interval [0,1]). Figure 1b illustrates an intermediate phase (EOD phase 0.52) which suggests a multipole field of lower amplitude (head and tail regions are negative, while the mid-body is positive).

**Figure 2:**
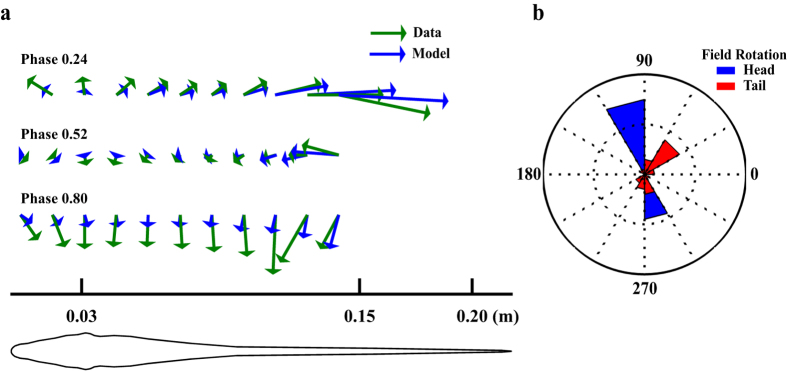
Analysis of electric field vectors. (**a**) Temporal evolution of the EOD field vectors along the rostrocaudal axis, at a distance 1.01 cm lateral to the body for the same EOD phases shown in Fig. 1. Electric field vectors are determined by the gradient of the local potential, and are illustrated by arrows of length and angle given by the field strength and orientation respectively (data in green, model in blue). (**b**) The distribution of electric field vector orientation over all 25 EOD phases. Measurements were made 2 cm lateral to the fish at rostrocaudal locations of −0.08 m (head, in blue) and 0.182 m (tail, in red); a rostrocaudal position of 0 m corresponds to the tip of the nose. In this angle histogram, bins are represented as wedges with an angular bin-width of 30°; the length of the wedge indicates the relative number of EOD phases having a field vector in the corresponding angular range.

To make accurate predictions for electric sensing (electric images)^13^,^20^,^30^,^31^,^32^, it is important to consider the electric field components as well as the potential. Note that we have fit the model using measurements of potential, and have not used the field components (see Methods). Nonetheless, we can compare the experimentally measured electric field vectors in space and time^10^,^11^ with those of our model. Figure 2a shows that our model captures the primary features of these dynamics (NRMSE<6%). In the front half of the body, the field vectors for model and data align well, with increasing differences towards the tail. The angle histogram (Fig. 2b) shows that the field vectors in the head region rotate very little over time (only two angular bins are filled in blue, indicating that the field vector is flipping between two directions); on the other hand, the field vectors in the tail region rotate through the entire cycle (most angular bins are filled in red, Fig. 2b; this head-tail difference has been noted previously^10^). One shortcoming of the model is in the field amplitudes, which are in general smaller than those measured experimentally. This is likely due to the 2D nature of our model. An application of Gauss’ law shows that the potential due to a point source in 3D will attenuate 1/r times faster than in 2D (see Appendix 1, Supplementary Information). Therefore, given that the electric field is the (negative) gradient of the voltage, over a region sufficiently close to the fish, the voltage will drop faster in 3D and result in an electric field with higher amplitude. This suggests that although our 2D model provides a good overall fit to the voltage map, the voltage gradient close to the fish is underestimated. There are also discrepancies in the angles of the electric field vectors. These angles will be influenced by body shape and other current-funneling properties, such as heterogeneities in the skin and body conductivity^18^. Any geometrical features not accounted for by the model could lead to small errors in electric field angle. We now describe the model solutions in more detail to provide insight into the dynamics of current generation by the electric organ.

### The electric organ current density

**Figure 3:**
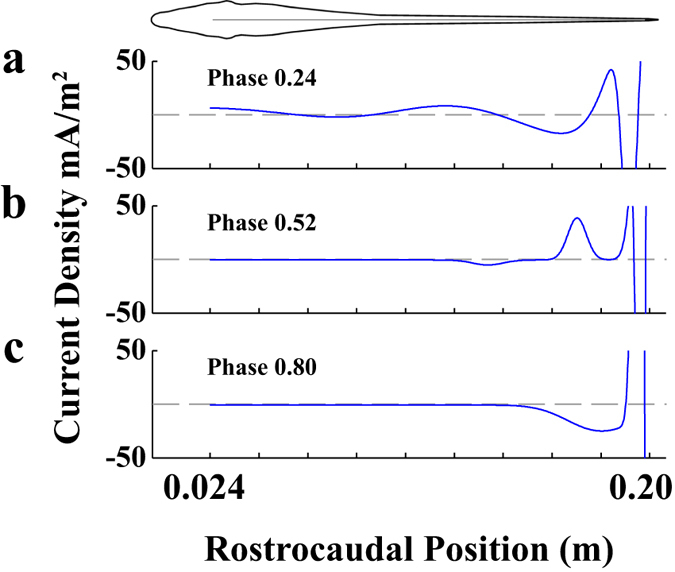
Examples of current density profiles along the electric organ. EOD phases are the same as those used in Fig. 1. Note that the scale of the current density was chosen to emphasize small changes in the mid-body, but results in clipping of the high amplitude current in the tail region. To emphasize the multiphasic nature of the EOD the gray dashed line indicates 0. (**a**) Head-positive EOD phase 0.24. (**b**) Intermediate phase EOD phase 0.52. (**c**) Head-negative EOD phase 0.80. A rostrocaudal position of 0 m corresponds to the tip of the nose, with 0.024 m denoting the rostral end of the electric organ.

**Figure 4:**
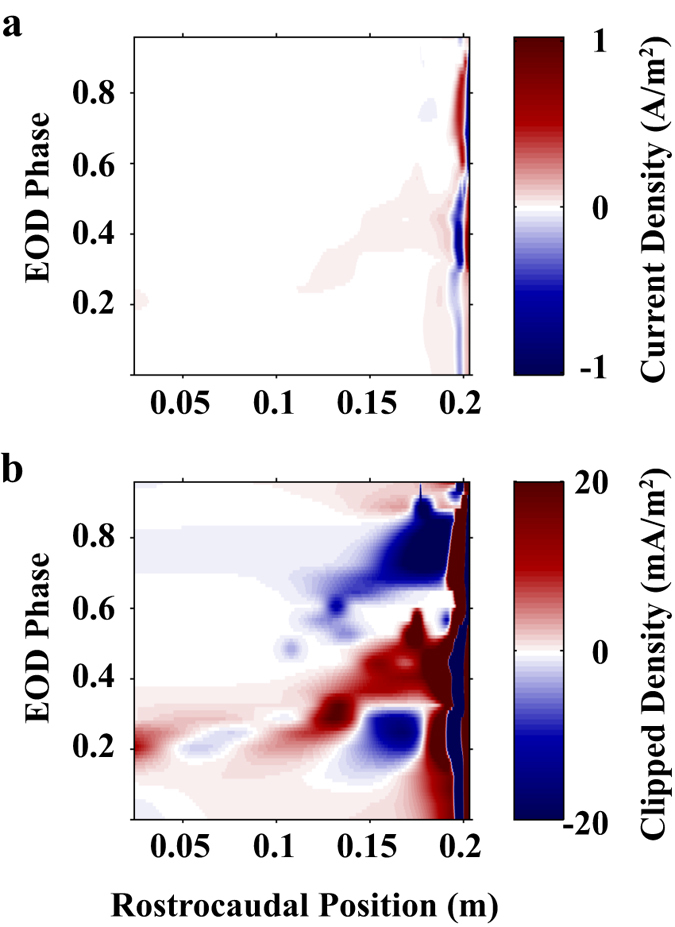
Spatiotemporal distribution of the EO current density. Positive current (red) is current efflux from the organ and negative current (blue) is current influx to the organ. (**a**) Full scale current density ±1 A/m^2^ (**b**) Current density color range clipped at ±20 mA/m^2^.

To obtain the potential maps and electric fields described above, we determined the current density along the boundary of the electric organ (EO) that minimized the RMSE between data and model for each of 25 EOD phases (see Methods). Fitting the model in this way provides a prediction for the spatiotemporal variation of current density on the EO. Figure 3 shows the current density profile along the EO resulting from these fits for the same three EOD phases used in Fig. 1 (EOD phase: 0.24, 0.52, and 0.80). The current density in the tail region is relatively large (~1 A/m^2^) so the y-axis is clipped to 50 mA/m^2^ in Fig. 3 to illustrate the lower amplitude variations in the mid-body. The unconstrained fitting process sometimes resulted in large current reversals in the tail region (due to overlapping Gaussian curves with opposite sign) but it is important to note that the net current in the tail is biphasic in time, switching from negative to positive over the EOD cycle. Integrating the current density over the caudal 1cm of the organ reveals this biphasic current profile (Fig. 3a: −0.09 mA/m; Fig. 3b: −0.25 mA/m; Fig. 3c: 0.74 mA/m). To visualize these dynamics more thoroughly, Fig. 4 shows the current density in space (rostrocaudal position along the electric organ) and time (EOD phase) as a color map. The disproportionately large currents in the tail are evident in Fig. 4a, whereas a similar expanded plot with current density clipped at 20mA/m^2^ (Fig. 4b) reveals a complex spatiotemporal pattern in the mid-body, with multiphasic temporal components (multiple positive peaks over the EOD cycle). Of particular note are the diagonal positive and negative patches, evidence of signal propagation along the EO. In the following subsections, we characterize these patterns using frequency-domain analyses over time and space.

### Spatiotemporal features of the EO current density

**Figure 5:**
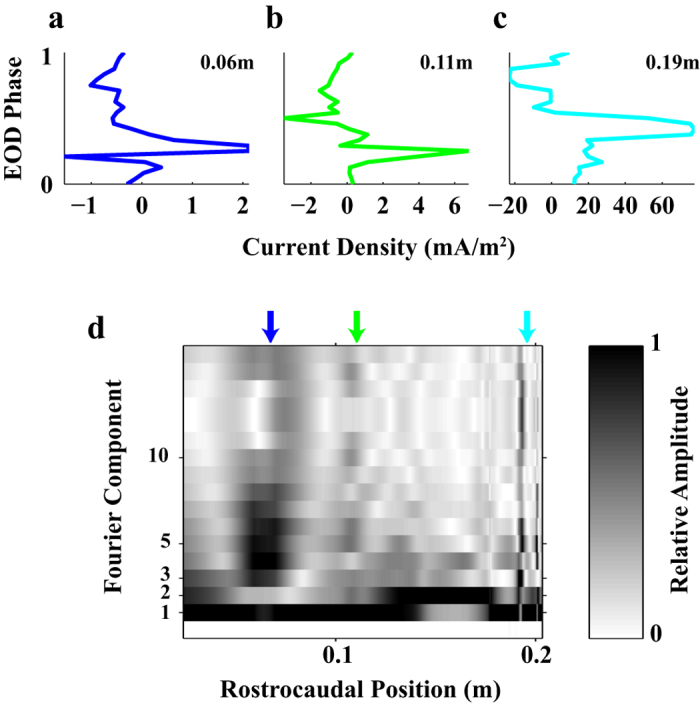
Temporal Fourier analysis of the EO current density. (**a–c**) Current density at three different rostralcaudal locations along the electric organ. (**d**) Amplitudes of the temporal Fourier components calculated at different locations along the electric organ. Amplitudes are normalized from [0,1] at each location. EOD phase is a normalized time between 0 and 1, corresponding to a complete EOD cycle. Color-coded current traces (**a–c**) are measured at locations indicated by colored arrows in d.

We first consider the temporal variations in current density at distinct locations along the EO. Figure 5(a–c) shows the time-varying currents at three different EO locations (indicated by distance from tip of the nose). The temporal diversity of these waveforms can be characterized using a Fourier analysis. The Fourier decomposition of the current waveform was calculated over the EOD cycle at each EO location. Figure 5d shows the normalized amplitude of different temporal frequency components as a function of the rostrocaudal location (amplitudes are normalized between 0 and 1 for comparison across the body). The amplitude of the fundamental (first Fourier component, equal to the EOD frequency) dominates over most of the EO, but the amplitude of higher harmonics differs along the EO. In the tail region (>0.16 m), the amplitudes of the 2^nd^ and 3^rd^ components (1^st^ and 2^nd^ harmonics) increase significantly. This is a reflection of the frequency doubling observed previously^11^ that results from the zero potential line sweeping back and forth over this region of the body. Higher harmonics are observed further caudal at the tip of the tail (where the current changes rapidly in time) and in the mid-body region (~0.06 m). In a pulse-type weakly electric fish, *Gymnotus carapo,* the electric organ comprises separate functional segments, each producing distinct waveforms^33^. It has been suggested^11^,^24^,^34^ that a similar type of segmental activation might occur in *A. leptorhynchus*, but the data shown here are more consistent with EO activation resembling a continuous travelling wave.

To further explore the travelling wave hypothesis, we next performed a Fourier analysis in space (i.e. rostrocaudally, along the EO). This involves considering a single point in time (single EOD phase) and then characterizing the spatial current profile over the EO by the amplitudes and phases of different spatial Fourier components. Repeating for all EOD phases provides a description of the evolution of EO activation. For reference, a pure oscillating dipole could be explained by a single spatial frequency component with a constant phase, but with amplitude varying in time. On the other hand, a phase that changes continuously in time is suggestive of a travelling wave.

**Figure 6:**
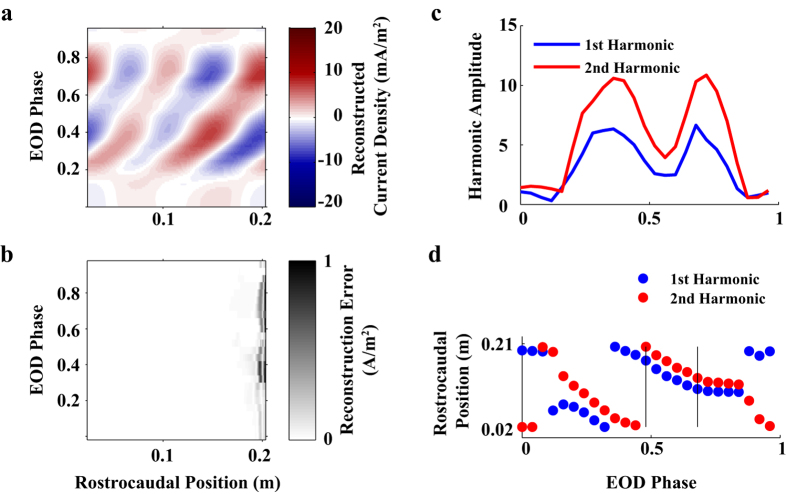
Spatial Fourier analysis of the EO current density. (**a**) Reconstruction of the EOD current with the 1^st^ and 2^nd^ spatial harmonics alone, NRMSE ~3.6%. (**b**), Absolute error map of the EOD reconstruction using the first two spatial harmonics (**c**) Amplitude of the 1^st^ and 2^nd^ spatial harmonics over time (EOD phase). (**d**) Rostralcaudal position of the 1^st^ and 2^nd^ spatial harmonics on the EO over EOD phase. Vertical lines indicate range over which propagation velocity was estimated.

We found that the primary features of the EO current density were well-represented (~3.8% RMSE) by only two spatial Fourier components (the 1^st^ and 2^nd^ harmonics). For clarification, the 1^st^ harmonic here is defined as the Fourier component with wavelength equal to the EO length. Figure 6a shows a reconstruction of the spatiotemporal pattern using only these two components (compare to Fig. 4b); with the exception of the tail region, the reconstruction error over the organ is small (Fig. 6b). The evolution of the amplitude and phase of each component along the EO is shown in Fig. 6c,d as a function of EOD phase. The amplitudes exhibit two peaks during the EOD cycle (Fig. 6c), but the phases of both components decrease systematically over the EOD cycle (Fig. 6d). The average velocity (measured over the region denoted by vertical lines in Fig. 6d) is approximately 210 m/s. These results not only support the hypothesis that EO activation comprises a continuous wave, but also suggest a propagation speed that is much faster than the 150m/s found in the fastest myelinated axons. The velocity of a wave is technically different than the velocities of its components, but given that the two main components have similar velocity, the velocity of the wave will be similar as well.

### Electric organ physiology

One prominent feature of the EOD in *A. leptorhynchus* is its large amplitude near the tail^10^,^11^,^24^. In some myogenic species with large cylindrical electrocytes, particular features of the EOD can be related to the anatomical characteristics of the EO and surrounding body tissues (see Gomez *et al.*^17^ for review). The looping structure of the thin fiber-like electrocytes in *A. leptorhynchus* makes this difficult. One possible explanation for the relatively large current in the tail-end is an increased number of electrocytes. If we assume a population of electrocytes that is homogeneous in electrical and anatomical properties, then the current flux at a particular location on the EO can provide a local estimate of electrocyte number. The current generated by an individual electrocyte is not known, but we can normalize the current flux at each location by the total current flux over the entire organ as a proxy of electrocyte density. Doing so suggests that the caudal 10% of the organ comprises 84% of the electrocytes, and that over the length of the organ, electrocyte density would increase by more than two orders of magnitude. While an increased electrocyte density has been suggested previously^35^, the massive increase suggested by this analysis seems unlikely.

A second possibility is that the electrocyte density is uniform and the increased potential in the tail is due to varying electrical properties of the body. A larger potential requires more current or increased resistance (V = IR). Increased resistance could arise from a high resistance membrane (as previously proposed^11^) or additional tissue near the tail, and could result in the voltage increase without an increase in the electrocyte number. A third possibility is that electrocytes increase in surface area in the caudal direction which would increase the current flux; however such detailed anatomical studies have not yet been performed. Finally, increased net current could also be achieved by higher levels of synchronization among electrocytes. Given their looped structure, current from electrocytes firing asynchronously could cancel destructively, leading to reduced net current flux from the EO.

## Discussion

In this study, we have used a finite-element approach to model the time-varying electric field of the wave-type electric fish, *A. leptorhynchus*. Models have been used previously to explore and describe the electric fields of various species^17^,^18^,^20^,^36^. These models fall into two main subgroups: analytic models and numerical models. Analytic methods, while demonstrating high computational efficiency are limited to systems where all equations have a known solution (which are available only for simple, isotropic shapes)^20^,^37^,^38^. Numerical methods can explore biologically relevant models involving more complex geometries^11^,^18^,^21^,^39^. Two dimensional (2D) models are often preferred as the number of equations is significantly reduced compared to 3D, placing much less demand on computer processing power and memory requirements. The boundary element method (BEM)^11^ provides an efficient approach to the 3D problem by solving equations only along boundaries of interest. An alternative to BEM models is an implementation of coupled resistors^39^, where each element in space is coupled orthogonally to surrounding elements and the resulting circuit problem can be solved efficiently by the application of Kirchhoff’s laws. Finite-element models (FEM) consider the realistic geometry and electrical properties of heterogeneous region of interest but can be computationally intensive.

Our study uses a 2D finite-element model that represents a significant improvement over previous models. Some previous models have considered the electric fields in both space and time^17^,^24^,^32^, but none have done so at the high spatiotemporal resolution considered here. Our approach, based on the extensive experimental data provided by Assad and coworkers^10^,^11^,^40^, has resulted in a detailed prediction of the spatiotemporal current density generated by the EO over the entire EOD cycle of a high-frequency wave-type fish. We have also provided support for travelling waves in the EO activation of *A. leptorhynchus.*

The spatial features of the electric field at a given point in time do not necessarily determine a unique EO current density. That said, we did not impose a continuity constraint during model fitting. Rather, for each EOD phase, all parameters were fit to the data independently of other phases. Remarkably, even without such a constraint, the fitted current solutions on the EO varied smoothly over EOD phase. While it is possible that the 8 Gaussians (24 parameters) used in our fits could over-fit the data, the resulting continuity of current density between phases implies that this is not the case. Overall, this suggests that the solutions, and thus our predictions, are robust.

The electric fields generated by weakly electric fish are often compared to those resulting from an oscillating dipole for which the polarity of the head and tail are of opposite sign. However, in many species, this analogy cannot explain the observed spatiotemporal complexities^40^. Using our model, we have predicted that these variations involve a current source that propagates down the EO towards the tail, traveling at ~210 m/s. Although this is much faster than action potential propagation in myelinated axons, speeds of up to 250 m/s have been predicted in another species of electric fish, *Brachyhypopomus*^41^. If the spinal axons innervating the EO cannot conduct action potentials at such high speeds, other mechanisms of signal generation in the EO must be considered. For example, the electrocytes in the EO may comprise a network of electrically-coupled oscillators^26^. Spinal inputs would then act to entrain the spontaneous signal, with no hard limit on the propagation speed of an electrical wave. Further studies must be performed to determine the electrophysiological properties of the fiber-like electrocytes in *A. leptorhynchus*.

One shortcoming of our model is that it considers only a 2D cross-section of the 3D electric field. It is thus possible that accounting for current flow outside of the horizontal plane will allow for a more parsimonious EO current density. However, the width (right-left) of these fish is small compared to the height (dorsal-ventral) making the lateral skin surface relatively flat; as a result, the current flow is predominantly in the horizontal plane, so our 2D approximation may not be a major problem. A simpler 2D model has been used to accurately locate weakly electric fish in shallow water using recordings from an electrode array^42^. Indeed, 2D models have also led to numerous insights about electrosensory processing, although for the accurate representation of the electric field in regions around the head and nose, 3D models are essential^10^,^36^,^43^. Nonetheless, future work should be focused not only on evaluating, anatomically and physiologically, the predictions of our model, but also on developing more anatomically-correct 3D models. Elaborating such electric field models by including the electrodynamics of neural and electrocyte activity (action potentials) will be another essential step. Such multi-physics approaches will provide a framework for understanding bioelectric field generation and localizing complex bioelectric current sources.

Weakly electric fish have been an effective model system in which to explore various aspects of bioelectric signalling. Most prominent is the work on electric field perturbations that provide a basis for electric imaging. The electric images produced by simple objects, investigated using both experimental and computational approaches, have provided novel insights into how electric fields can be used for object localization and identification^13^,^36^. Recently, these developments have been implemented in autonomous robots^44^,^45^. Future work could also suggest novel ways to improve electrical impedance imaging currently used in a clinical context^45^,^46^. Our model could facilitate such work by the systematic manipulation of parameters involved in electric field geometry and sensing surface (body shape and electrical properties) to optimize particular features of the electric image.

The problem of bioelectric source identification has received less attention in the electric fish literature^24^,^42^. The present study is the first to make a detailed prediction of the current density along the electric organ. The experimental accessibility and well-defined anatomical structures in electric fish make our predictions readily testable. Because this is one of a general class of problems involving bioelectric current sources, an efficient cycle of model prediction and experiment in electric fish will likely lead to broadly applicable insights. The interpretation of EEG recordings is of particular relevance in this context^47^. Identifying the signal sources underlying brain activity could in principle be confounded by the type of high-speed signal propagation we have described in the electric organ. Understanding the biophysical nature of high-speed signalling in electric fish should thus provide a framework for studying analogous phenomenon in more complex nervous systems.

## Methods

### Electric Field Modeling

The finite-element approach presented here to model the electric potential of *Apteronotus leptorhynchus* (brown ghost knife fish) is based on previous work focused on a single phase of the EOD cycle^18^,^30^ (but also see reference^32^ for the description of two additional phases). Here, we include 25 different phases of the EOD cycle, based on data provided by Chris Assad and Brian Rasnow^10^,^11^,^40^. Briefly, the fish body, skin and electric organ in the horizontal plane are described by a 2D finite-element model. The model set-up mimicked the experimental setup^11^. The fish (21 cm in length; EOD frequency of 810 Hz) was centered in a 60 cm × 60 cm tank filled with water (conductivity of 230 μS/cm). A ground electrode (silver wire, 250 μm diameter) was placed in a corner of the tank closest to the head^11^,^18^. The body outline was based on experimental data, but made symmetrical (right-left) with the opercula removed. Body conductivity was 0.356 S/m^18^ which is consistent with experimental results found in *Gnathonemus petersii*^48^. A 100 μm skin layer was used, with skin conductivity varying from 0.25 mS/m to 2.5 mS/m (from head to tail) as in Babineau *et al.*^18^.

In order to calculate the electric potential (and electric field), Poisson’s equation was solved in three dimensions using COMSOL Multiphysics v5.0 (COMSOL Inc., Burlington, MA):


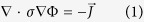


where 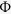 is the electric potential, σ is the electrical conductivity, and *J* is the total current flux through an element (note that current flux is used to denote current entering and leaving an element, but it is a current density, A/m^2^). In order to restrict the model to two dimensions, the conductivity along the dorsoventral axis was set to 0 S/m. For the low frequencies used by the fish (<10 kHz), capacitive effects are negligible and the fish body is mostly resistive^8^,^23^.

### Electric Organ Modeling

The electric organ (EO), modelled as a caricature of the organ described by Bennett^26^, comprised a single rectangle (17.97 × 0.025 cm) positioned 2.36 cm from the tip of the nose and symmetric about the rostrocaudal axis. The electric organ acts as a current source^26^, and has been described in different ways in previous models^11^,^18^,^20^. Here, extending Babineau’s approach^18^, we define the current density along the organ as a sum of Gaussians (eq. 2):


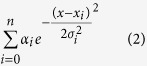


where α_i_ is the amplitude, σ_i_ is the standard deviation and *x*_*i*_ is the mean of the i^th^ Gaussian (i = 1,…,n). Babineau *et al.*^18^ used two Gaussians (n = 2) to describe a single EOD phase. Visual inspection of the EOD voltage along the fish over all phases suggested there would be four to five peaks in the EO current density. To ensure that the model captured additional detail, we chose eight Gaussians (n = 8) for the following analyses. Following model optimization at each phase, the amplitude coefficients of each fitted Gaussian were rank-sorted; in all cases but one, the value of the smallest coefficient was less than 0.1% of maximum, indicating that very little would be gained by adding more Gaussians (for the one outlier phase, this value was 5% of maximum). However, we are not suggesting that there is any physiological significance to our choice of eight (we use a subsequent Fourier analysis to address this issue, see Results). The lateral boundaries of the organ (long dimension of the rectangle) were defined as current sources, where the initial conditions for the current is normal flow, but at steady state there are no orientation conditions. The “ends” of the organ (short dimension of the rectangle) have no defined current nor do they have a restriction on current passing through them.

### Model Optimization

The experimental data from Assad and Rasnow quantify the electric field and potential over 25 phases of the EOD cycle (phase is indicated as a fraction of cycle period, varying from 0 to 1). At each phase, measurements were made with varying spatial resolution on one side of the fish from 8 cm rostral to the head, to 5.2 cm caudal to the tail and up to 14 cm lateral to the fish (for details, see references^11^,^18^). These data points were interpolated to 2 mm resolution within 1 cm of the fish and to 6 mm resolution from 1 cm to 5 cm lateral to the fish (733 total points). Varying the spatial resolution in this manner implicitly assigns a higher weight to measurements closer to the fish during model optimization.

For each of the 25 phases in the EOD cycle, the parameters for the EO current density (eight Gaussians with three parameters each; eq. 2) were chosen to minimize the root-mean squared error (RMSE) between the model and data:


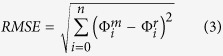


where 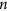 is the number of data points, and 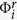 and 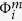 are the experimental measurements and model predictions (respectively) for the electric potential at the 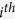 point in space. To minimize the RMSE, the SNOPT algorithm (a gradient descent algorithm) was implemented in COMSOL^49^. For initial conditions, all Gaussian amplitudes and means were set to 0 and the standard deviations were set to 0.01 m; during fitting, no constraints were placed on parameter values so each Gaussian could change amplitude, width, and move up or down the EO. Due to the large number of parameters, if the RMSE changed very slowly by the 500^th^ iteration, the fitting procedure was stopped for that phase. The goodness of fit is reported as the normalized RMSE (NRMSE):


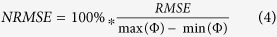


where 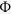 is the voltage given by the model at all locations for a single EOD phase, and RMSE is the root-mean-squared error for the fit in question. Each phase was treated independently, with no constraints on smoothness between phases. At this stage, we have not yet considered the biophysical details of how this EO current is generated.

Current conservation was evaluated by calculating the net current flow (∑ *I*) across all EO boundaries over the entire EOD cycle. For the solutions presented in this paper, net current was −0.36 A/m^2^, less than 0.1% of the total current flux 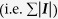. This is consistent with experimental observations that show no DC-offset in EOD recordings of this species of weakly electric fish^26^.

## Additional Information

**How to cite this article**: Shifman, A. R. *et al.* Ultrafast traveling wave dominates the electric organ discharge of *Apteronotus leptorhynchus:* an inverse modelling study. *Sci. Rep.* **5**, 15780; doi: 10.1038/srep15780 (2015).

## Supplementary Material

Supplementary Information
