# Supplementary material for: Ultrafast traveling wave dominates the electric organ discharge of Apteronotus leptorhynchus: an inverse modelling study
Source: Sci Rep. 2015 Oct 30;5:15780. doi: 10.1038/srep15780 (PMC4626797; doi:10.1038/srep15780)
Supplement: Supplementary Information [file srep15780-s1.pdf]

Ultrafast traveling wave dominates the electric organ discharge of *Apteronotus leptorhynchus*: an inverse modelling study

Aaron R. Shifman[1,2], Andre Longtin[2,3], John E. Lewis[1,2,*]

Appendix 1

Fall-off of the electric field in 2D and 3D

Assuming a point charge in an infinite perfectly symmetric region

$$\Phi = \oint_S \vec{E} * d\vec{A} = \frac{q}{\epsilon_0} \qquad (Gauss' law)$$

where the integral is over the bounded closed surface S containing the net charge *q*.

In 2D, *dA* is replaced with *dr* (as a circle can be defined but not a sphere) and S is now the perimeter of the closed curve enclosing the charge Q. For the simple case of a charge at the center of a circle we have

$$E \oint_S d\vec{r} = E * 2\pi r = \frac{q}{\epsilon_0} \Rightarrow E = \frac{q}{r * 2\pi\epsilon_0} \Rightarrow E \propto \frac{1}{r}$$

In 3D

$$E \oint_S d\vec{A} = E * 4\pi r^2 = \frac{q}{\epsilon_0} \Rightarrow E = \frac{q}{r^2 * 4\pi\epsilon_0} \Rightarrow E \propto \frac{1}{r^2}$$

Therefore in 3D the field will fall off as the square of the distance whereas in 2D the field will fall off with the distance.
